# Neutrophil-to-Lymphocyte Ratio and Platelet-to-Lymphocyte Ratio are Predictors of Heart Failure

**DOI:** 10.5935/abc.20150126

**Published:** 2015-12

**Authors:** Erdal Durmus, Tarik Kivrak, Fethullah Gerin, Murat Sunbul, Ibrahim Sari, Okan Erdogan

**Affiliations:** 1 Silifke State Hospital, Cardiology Clinic, Mersin, Turkey; 2 Sivas Numune Hospital, Cardiology Clinic, Sivas, Turkey; 3 Central Laboratory of Public Health, Department of Clinical Biochemistry, Istanbul, Turkey; 4 Marmara University Faculty of Medicine, Department of Cardiology, Istanbul, Turkey

**Keywords:** Heart Failure / blood; Heart Failure / diagnosis, Multivariate Analysis, Neutrophils / cytology, Leukocyte Count, Lymphocyte Count

## Abstract

**Background:**

Neutrophil-to-lymphocyte ratio (NLR) and platelet-to-lymphocyte ratio (PLR) are inflammatory markers used as prognostic factors in various diseases. The aims of this study were to compare the PLR and the NLR of heart failure (HF) patients with those of age-sex matched controls, to evaluate the predictive value of those markers in detecting HF, and to demonstrate the effect of NLR and PLR on mortality in HF patients during follow-up.

**Methods:**

This study included 56 HF patients and 40 controls without HF. All subjects underwent transthoracic echocardiography to evaluate cardiac functions. The NLR and the PLR were calculated as the ratio of neutrophil count to lymphocyte count and as the ratio of platelet count to lymphocyte count, respectively. All HF patients were followed after their discharge from the hospital to evaluate mortality, cerebrovascular events, and re-hospitalization.

**Results:**

The NLR and the PLR of HF patients were significantly higher compared to those of the controls (p < 0.01). There was an inverse correlation between the NLR and the left ventricular ejection fraction of the study population (r: -0.409, p < 0.001). The best cut-off value of NLR to predict HF was 3.0, with 86.3% sensitivity and 77.5% specificity, and the best cut-off value of PLR to predict HF was 137.3, with 70% sensitivity and 60% specificity. Only NLR was an independent predictor of mortality in HF patients. A cut-off value of 5.1 for NLR can predict death in HF patients with 75% sensitivity and 62% specificity during a 12.8-month follow-up period on average.

**Conclusion:**

NLR and PLR were higher in HF patients than in age-sex matched controls. However, NLR and PLR were not sufficient to establish a diagnosis of HF. NLR can be used to predict mortality during the follow-up of HF patients.

## Introduction

The prevalence of heart failure (HF) is increasing due to the aging of the population and the decreased cardiac mortality and morbidity related to modern treatment approaches worldwide. The prevalence of HF is approximately 10% in patients aged over 70 years^1^. Despite advanced treatment approaches, mortality rates are still high. Moreover, the need for long-term care of HF patients constitutes a difficult situation for caregivers and relatives of patients.

Previous studies have shown that inflammation plays an important role in the initiation and progression of cardiovascular diseases (CVD)^2-5^. Chronic inflammation is also more common in HF patients^6^. White blood cells (WBCs) and their subtypes are associated with increased cardiovascular risk factors^7-9^. Neutrophil-to-lymphocyte ratio (NLR) and platelet-to-lymphocyte ratio (PLR) are novel inflammatory biomarkers used as prognostic factors in various diseases^6,10,11^. Previous studies have demonstrated that a higher NLR is associated with a higher mortality rate in those with coronary artery disease (CAD)^5,6,10^. A study carried out by Uthamalingam et al^6^ showed that a higher NLR is associated with higher mortality in HF patients. However, to the best of our knowledge, there is no data about the significance of the PLR in HF patients in the literature. Comparison of the PLR of HF patients with those of age-sex matched healthy controls has not been studied previously. Therefore, the first aim of this study was to compare the PLR and the NLR of HF patients with those of age-sex matched controls. The second aim was to evaluate the predictive value of NLR and PLR in detecting HF. The third aim was to demonstrate the effect of the PLR and the NLR on mortality in HF patients during follow-up.

## Methods

### Study population

Between June 2012 and October 2013, 71 patients diagnosed with decompensated HF were evaluated for study enrollment. A decompensated HF diagnosis was made if the patients had one of the typical HF symptoms, such as dyspnea at rest, orthopnea, or paroxysmal nocturnal dyspnea, and one of the typical HF signs, such as bilateral rales, pretibial edema, jugular venous distension, or a n-terminal pro brain natriuretic peptide (NT-pro BNP) level more than two times the upper limit of normal values depending on age group and according to current HF recommendations^1^. If patients had no symptoms or signs of HF despite a reduced ejection fraction (EF), they were not diagnosed as having decompensated HF. The plasma level of NT-pro BNP was measured using an Elecsys 2010 (Roche) device and an electrochemiluminescence immunoassay method. Patients with renal failure, acute and chronic infection, acute coronary syndromes, or connective tissue disease were excluded from the study. After the exclusion criteria were applied, 56 decompensated HF patients were included in the study. Forty patients who were admitted to our cardiology clinic and who were proven to have no cardiac abnormality after a complete cardiac evaluation, including a physical examination, electrocardiography, and echocardiography, and who had not been previously hospitalized for HF were included in the study as a control group. The whole study population was evaluated for the presence of hypertension (HT), hyperlipidemia (HL), and diabetes mellitus (DM). Hypertension was defined as systolic and/or diastolic blood pressure ≥ 140 /90 mm Hg, previously diagnosed HT, or the use of any antihypertensive medication. Diabetes mellitus was defined as fasting plasma glucose levels > 126 mg/dL in ≥ 2 measurements, previously diagnosed DM, or the use of antidiabetic medications, such as oral antidiabetic agents or insulin. Hyperlipidemia was defined as serum total cholesterol ≥ 200 mg/dL, serum triglyceride ≥ 150 mg/dL, low-density lipoprotein cholesterol ≥ 130 mg/dL, previously diagnosed HL, or if a patient had to use lipid-lowering medication.

Complete blood cell counts, which included total WBCs, neutrophils, lymphocytes, and platelets, were obtained at the time of admission. The NLR and the PLR were calculated as the ratio of neutrophil count to lymphocyte count and as the ratio of platelet count to lymphocyte count, respectively.

All decompensated HF patients who clinically improved with treatment were followed up after discharge. The data about death, cerebrovascular events, and re-hospitalization were gathered via telephone. The study was approved by the local ethics committee, and written informed consent was obtained from all participants.

### Assessment of standard transthoracic echocardiography

All patients underwent complete two-dimensional (2D) transthoracic echocardiography with a commercially available echocardiography device (Vivid 7, GE Vingmed Ultrasound AS, Horten, Norway) operated by a single experienced cardiologist. Data acquisition was performed with a 3.5-MHz transducer at a depth of 16 cm in the parasternal and apical views (standard parasternal short-axis view at the midventricular level, apical long-axis, two-chamber and four-chamber images), and a color Doppler frame scanning rate of 100-140 Hz was used for color time delay and integration (TDI) images. Echocardiographic parameters were measured according to the recent guidelines of the American Society of Echocardiography, and the left ventricular (LV) EF was calculated using the biplane Simpson method^12^.

### Statistical analysis

For the statistical analysis, we used the Statistical Package for the Social Sciences (SPSS) 15.0 for Windows. Categorical variables were defined as a percentage, and comparisons were made using the chi-square test. Quantitative variables were expressed as mean ± standard deviation, and, for the comparison of variables between the two groups, Student *t* test (parametric distributed parameters) and Mann-Whitney U test (for the parameters showing the nonparametric distribution) were used. To compare more than two groups, a Kruskal-Wallis test (nonparametric distributed data) was used, and the results were assessed using post-hoc analysis. Correlation analyses were performed using a Spearman correlation test. Receiver operating characteristic (ROC) analysis was used to assess the ability of the NLR and the PLR to predict the presence of HF. For multivariate analysis to evaluate the effects of independent parameters on the presence of HF and mortality, we performed logistic regression analysis. A p value < 0.05 was accepted as statistically significant.

## Results

The study population consisted of 56 patients with decompensated HF [40 with reduced EF (< 50%), 16 with preserved EF (> 50%)] and 40 age-sex matched controls without HF. The baseline characteristics and echocardiographic parameters of the HF patients and controls are shown in Table 1. There were no statistically significant differences in age and sex between the two groups (p = 0.20, p = 0.780, respectively). Atrial fibrillation (AF), DM, HT, and HL were observed to be more common in the HF group compared to the controls. Left ventricular diameters and left atrial area were significantly larger in HF patients, and the LVEF was significantly lower in HF patients compared to the controls. The medications of HF patients and controls are listed in Table 2.

**Table 1:** Comparison of baseline characteristics and conventional echocardiographic parameters of study population

	**Patients (n = 56)**	**Controls (n = 40)**	**p**
Age (years)	67.5 ± 12.6	64.6 ± 8.5	0.20**
Sex (male, n)	32	24	0.780*
AF (n)	33	0	< 0.001*
DM (n)	28	6	< 0.001*
HT (n)	49	19	< 0.001*
HL (n)	18	3	0.004*
EF (%)	40.8 ± 12.8	65.4 ± 4.9	< 0.001
LVEDD (mm)	55.0 ± 14.5	46.0 ± 3.2	< 0.001
LVESD (mm)	43.9 ± 15.4	29.0 ± 3.1	< 0.001
LAA (cm^2^)	25.6 ± 10.1	15.5 ± 2.2	< 0.001

Data are presented as mean ± standard deviation or number of patients. AF: Atrial fibrillation; DM: Diabetes Mellitus; HT: Hypertension; HL: Hyperlipidemia; EF: Ejection fraction; LVEDD: Left ventricle end-diastolic diameter; LVESD: Left ventricle end-systolic diameter; LAA: Left atrial area.

* Chi-square test was used,

** Student t test was used, others were evaluated with Mann-Whitney U test.

**Table 2:** Current medications of heart failure patients and controls

**Group of drugs**	**Patients**	**Controls**	**p**
Diuretics (%)	40 (71.4)	0 (0)	< 0.001
ACEI-ARB (%)	32 (57.1)	19 (47.5)	0.351
Calcium channel blockers (%)	21 (37.5)	8(20)	0.66
Beta-blocker (%)	33 (58.9)	2(5)	< 0.001
Digoxin (%)	15 (26.8)	0 (0)	< 0.001
Nitrates (%)	5 (8.9)	(0)	< 0.001

Data are presented as number and percentage of patients. ACEI: Angiotensin-converting-enzyme inhibitors; ARB: Angiotensin receptor blockers.

**Figure 1:**
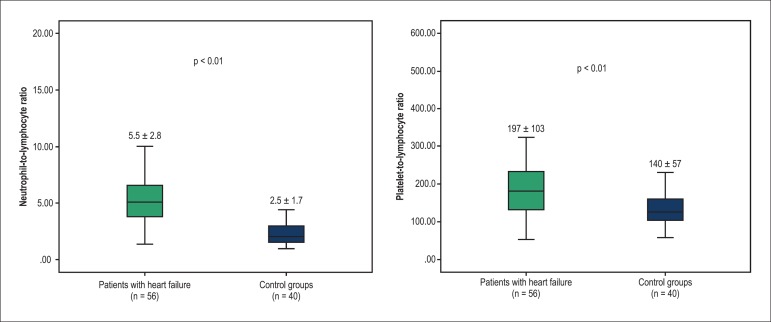
Comparison of neutrophil-to-lymphocyte ratio and platelet-to-lymphocyte ratio between two groups.

**Figure 2:**
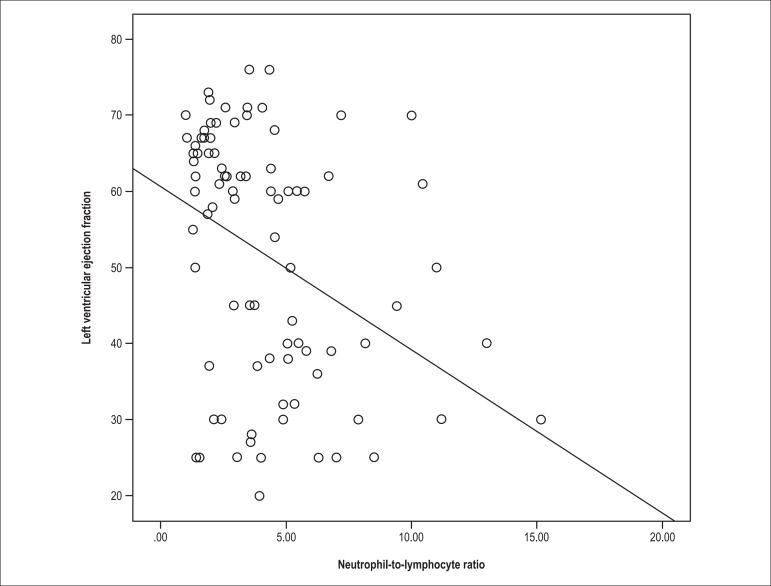
Correlation analysis of neutrophil-to-lymphocyte ratio with left ventricle ejection fraction.

**Figure 3:**
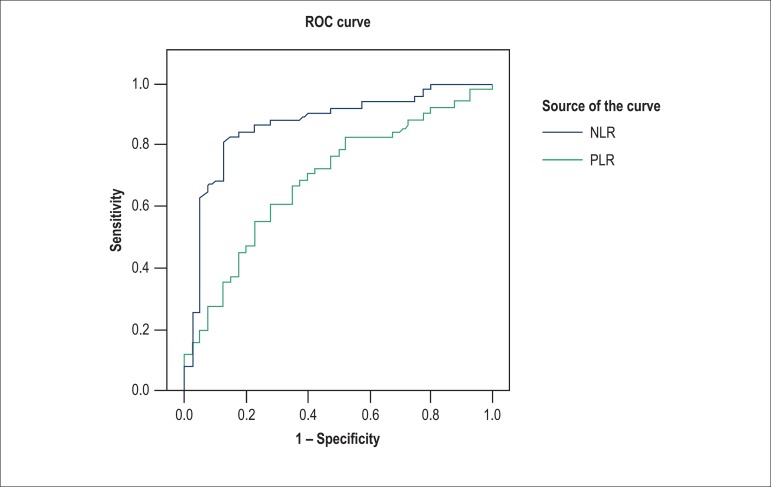
Receiver Operating Characteristic (ROC) curve analysis of neutrophil-to-lymphocyte ratio (NLR) and platelet-to-lymphocyte ratio (PLR) to predict presence of heart failure.

A comparison of laboratory findings between the two groups is shown in Table 3. White blood cell counts were similar in both groups, whereas the NLR (5.5 ± 2.8 vs. 2.5 ± 1.7, p < 0.01) and the PLR (197 ± 103 vs. 140 ± 57, p < 0.01) of the HF patients were significantly higher than those of the controls (Figure 1). We also compared the NLR and the PLR of HF patients to check for the presence of cardiovascular risk factors including AF, DM, HT, and HL (Table 4). The NLR and the PLR of AF patients were higher than those of the patients without AF. A correlation analysis performed using a Spearman test revealed that there was an inverse correlation between the NLR and the LV EF in the study population (r: -0.409, p < 0.001) (Figure 2). The NLR also correlated positively with the left atrium area (r: 0.496, p < 0.001). After ROC analysis, the best cut-off value of the NLR to predict the presence of HF was 3.0 with 86.3% sensitivity and 77.5% specificity (AUC: 0.868, p < 0.001), and the best cut-off value of the PLR to predict HF was 137.3 with 70% sensitivity and 60% specificity (AUC: 0.689, p = 0.004) (Figure 3).

**Table 3:** Comparison of laboratory findings between two groups

	**Patients (n = 56)**	**Controls (n = 40)**	**p**
Hemoglobin (gr/dL)	11.7 ± 1.6	13.3 ± 1.8	< 0.001*
WBC (10^3^/µL)	7.6 ± 2.2	7.2 ± 1.6	0.322**
Neutrophils (10^3^/µL)	5.6 ± 1.8	4.3 ± 1.3	< 0.001**
Lymphocytes (10^3^/µL)	1.2 ± 0.7	2.0 ± 0.7	< 0.001**
Platelets (10^3^/µL)	203 ± 68	254 ± 63	< 0.001*
NLR	5.5 ± 2.8	2.5 ± 1.7	< 0.001**
PLR	197± 103	140 ± 57	0.001**

Data are presented as mean ± standard deviation. WBC: White blood cell; NLR: Neutrophil-to-lymphocyte ratio; PLR: Platelet-to-lymphocyte ratio. Parameters evaluated with

* Student t test and

** Mann-Whitney U test.

**Table 4:** Comparison of neutrophil-to-lymphocyte ratio (NLR) and platelet-to-lymphocyte ratio (PLR) levels according to presence of cardiovascular risk factors in patients with heart failure

	NLR		p	PLR		p
	Yes	No		Yes	No	
AF	5.38 (2.80)	2.64 (2.55)	< 0.001	194.3 (123.7)	135.2 (77.4)	0.006
DM	4.27 (2.85)	3.15 (3.52)	0.188	151.1 (222.2)	153.3 (120)	0.957
HT	3.95 (3.22)	2.56 (3.10)	0.141	153.3 (105.4)	140.5 (102.1)	0.314
HL	4.54 (3.25)	3.50 (3.40)	0.154	134.2 (104.7)	154.8 (112.5)	0.349

Data are presented as median (interquartile range). AF: Atrial fibrillation; DM: Diabetes mellitus; HT: Hypertension; HL: Hyperlipidemia.

After discharge from hospital, we acquired information about the HF patients via telephone. The average follow-up duration of HF patients was 12.8 ± 7.6 months (min-max: 1-26 months). The incidence of death, cerebrovascular events, and re-hospitalization of HF patients were 17.9%, 3.6%, and 32.1%, respectively. The cut-off value of 5.1 for the NLR can predict mortality in HF patients with 75% sensitivity and 62% specificity (AUC: 0.730, p = 0.045) during a 12.8-month follow-up period on average^13^. Based on multivariate logistic regression analysis, only the NLR was an independent predictor of mortality in HF patients (Table 5).

**Table 5:** Multivariate logistic regression analysis to determine the independent predictors of mortality in heart failure

	**Odds Ratio**	**95% Confidence Interval**	**p**
Age (years)	1.027	0.928 - 1.136	0.608
Sex (male)	2.772	0.386 - 19.895	0.311
EF (%)	0.944	0.867 - 1.027	0.179
NLR	1.680	1.013 - 2.786	0.045
PLR	0.993	0.976 - 1.010	0.407

NLR: Neutrophil-to-lymphocyte ratio; PLR: Platelet-to-lymphocyte ratio; EF: Ejection fraction.

## Discussion

In this study, we demonstrated that the NLR and the PLR were higher in HF patients compared to age-sex matched controls. To the best of our knowledge, this is the first study to demonstrate that the PLR levels of HF patients are higher than those of controls. Moreover, the NLR was inversely correlated with the EF and is also an independent predictor of HF mortality. During the follow-up of HF patients, the NLR can be used to predict mortality.

Chronic inflammation is observed to be more common in chronic diseases, such as cancers, DM, HT, connective tissue disease, and chronic kidney disease^14-18^. Increased levels of inflammation are also associated with a poor prognosis in CAD^4^. Inflammatory reactions play a pivotal role in the development of HF^19,20^. White blood cells and their subtypes are remarkable inflammatory markers in CVD. As a result of inflammatory stimulus, leukocytes release many inflammatory cytokines, such as TNF-α , IL-6, and CRP, as well as some proteolytic enzymes. These pro-inflammatory cytokines have destructive effects on the myocardium, resulting in decreased LV function and HF^21-24^. Previous studies have shown that higher levels of pro-inflammatory cytokines may lead to myocardial remodeling and cardiac arrhythmia^25,26^. Lymphocytes play an important role in healing by modulating mononuclear cell phenotypes and inducing the tissue inhibitor of metalloproteinase-1 expression^27^. Lymphopenia is seen to be more common in stressful conditions such as HF due to the activation of the hypothalamic-pituitary-adrenal axis. The activation of this axis leads to cortisol secretion, and increased cortisol levels result in a decrease in the relative concentration of lymphocytes^28,29^. Lymphopenia is an independent prognostic factor and is also associated with decreased survival in patients with HF^29,30^.

Heart failure is a chronic disease with high mortality rates. The estimated one-year mortality rate is more than 20%^6^. The most powerful predictors of mortality are older age (more than 60), the presence of DM, and a lower LVEF^13^. Because of poor prognosis in HF, determining the prognostic factors is important for these patients. In a previous study of acute decompensated HF patients, higher NLR values were associated with a higher mortality rate, and the ability of the NLR to predict mortality was superior to that of the neutrophil count, the total WBC count, and a relative low lymphocyte count^6^. Therefore, an increased NLR in HF was associated with poor prognosis. In HF patients, despite similar total WBC counts compared to age-sex matched controls, the NLR was changing in favor of neutrophils. In this study, we proved that the NLR of HF patients was significantly higher than that of controls. The cut-off values of 3.0 and 5.1 can be used to predict the presence of HF and mortality, respectively. A study of HF patients demonstrated that after levosimendan infusion therapy, patients with higher NLRs had a higher mortality rate compared to patients with lower NLRs, and the 5.5 cut-off value of the NLR was determined as an effective cut-off point for predicting in-hospital mortality^31^. Previous studies have demonstrated that higher platelet and lower lymphocyte counts are associated with poor cardiovascular outcomes. High PLR is associated with a poorer prognosis in various disease states, such as several cancers and CAD^32-34^. However, there has been no available data about the PLR in HF patients until now. This is the first study to demonstrate that the PLR levels of HF patients are higher than those of controls. The cut-off value 137.3 can be used to predict the presence of HF.

### Study limitations

Our study had some limitations. One was the small sample size. This might be why the power of the study was lower. We did not observe a significant correlation between the PLR and the LVEF for that reason. Due to the increased range of the PLR level and the small sample size, the sensitivity and specificity levels were too low to predict HF (70% and 60%, respectively). Therefore, the cut-off point value was close to the mean PLR level in the control group. We did not measure the level of inflammatory markers, such as TNF-α , IL-6, and CRP. Therefore, we did not compare the prognostic value of the NLR and those inflammatory factors. In addition, this was an observational and nonrandomized study; it can be accepted as a prototype of further prospective and randomized studies to compare the effect of the NLR on cardiovascular mortality.

## Conclusions

The NLR and the PLR of HF patients were higher than those of the age-sex matched controls. However, the NLR and the PLR were not sufficient to establish a diagnosis of HF. The NLR may be used in the HF patient follow-up to predict mortality.
